# Supplementary figures and images for: The impact of long-term conditions on disability-free life expectancy: A systematic review
Source: PLOS Glob Public Health. 2022 Aug 5;2(8):e0000745. doi: 10.1371/journal.pgph.0000745 (PMC10021208; doi:10.1371/journal.pgph.0000745)

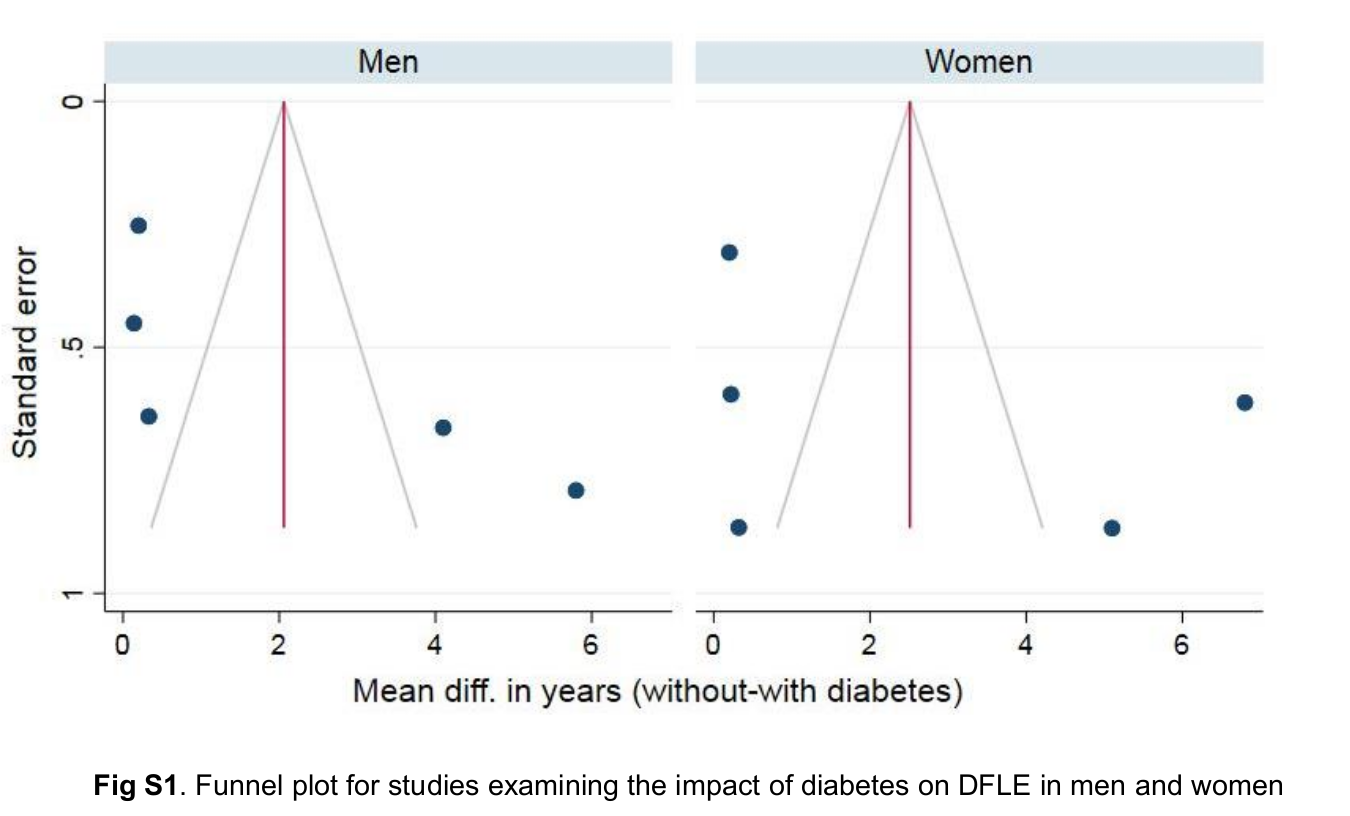

Supplement: S1 Fig — (TIF) [file pgph.0000745.s002.tif]

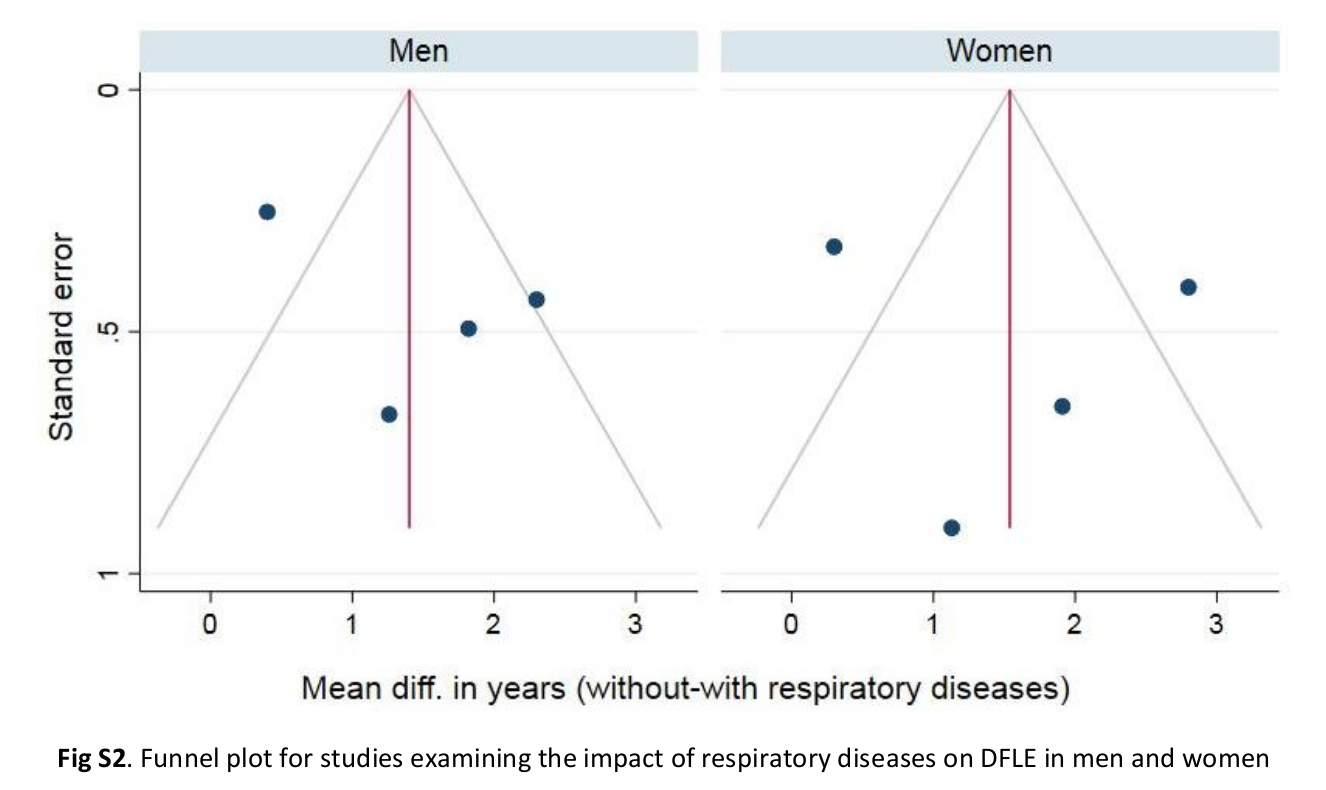

Supplement: S2 Fig — (TIF) [file pgph.0000745.s003.tif]

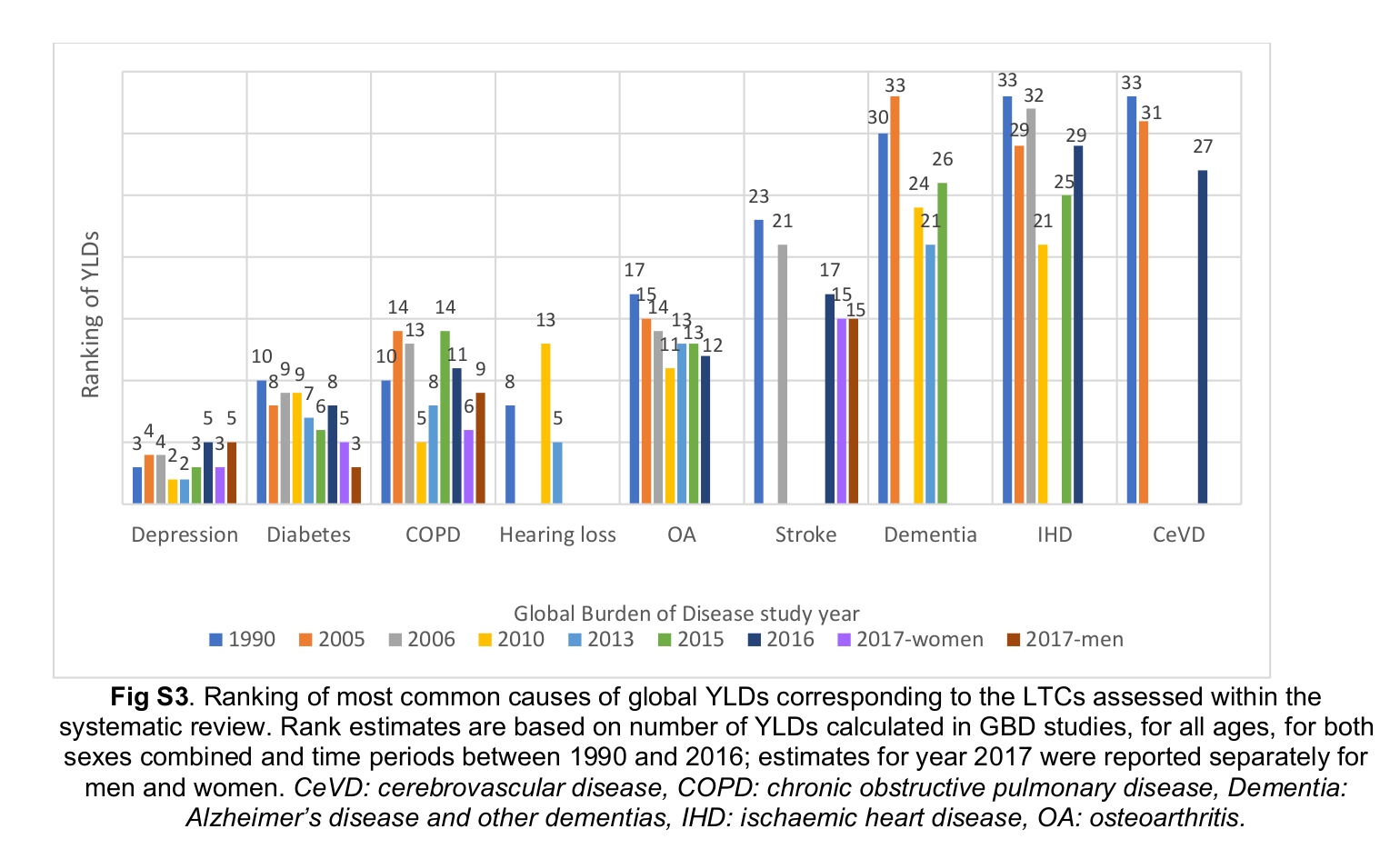

Supplement: S3 Fig — (TIF) [file pgph.0000745.s004.tif]

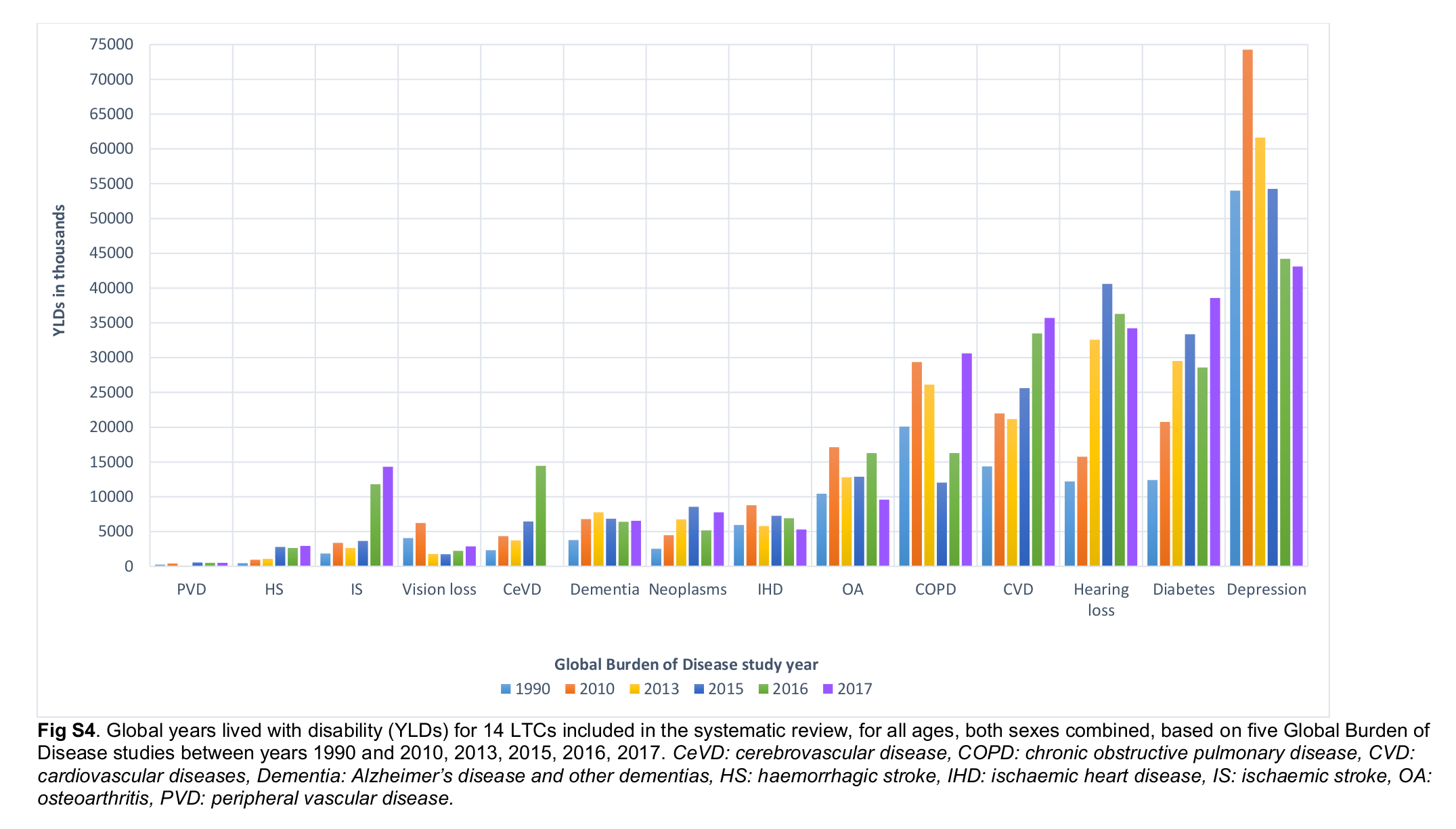

Supplement: S4 Fig — (TIF) [file pgph.0000745.s005.tif]
