# Supplementary material for: The impact of long-term conditions on disability-free life expectancy: A systematic review
Source: PLOS Glob Public Health. 2022 Aug 5;2(8):e0000745. doi: 10.1371/journal.pgph.0000745 (PMC10021208; doi:10.1371/journal.pgph.0000745)
Supplement: S1 Table — (DOCX) [file pgph.0000745.s006.docx]

**S1 Table.** Quality assessment for 13 cross-sectional studies (adapted version of JBI checklist supplemented with Freedman criteria)

| Study (year) | Sample frame appropriate to address the target population? | Study participants recruited in an appropriate way? | Sample size adequate? | Study subjects and setting described in detail? | Data analysis conducted with sufficient coverage of the identified sample? | Valid methods used for the identification of the condition? | **Was the response rate adequate, and if not, was the low response rate managed appropriately?** | Condition measured in a standard, reliable way for all participants? | **Quality of outcome measures (Detailed self-reports; Global self-reports; none)** | **Proxy, % (<10; 10-20; >20)** | **Missing data, % (<5; 5-10; >10)** |
| --- | --- | --- | --- | --- | --- | --- | --- | --- | --- | --- | --- |
| Bronnum-Hansen (2006)[3] | Yes | Yes | Yes | No | No | No | Unclear | Yes | Fair | Unclear | Unclear |
| Campolina (2013)[12] | Yes | Yes | Yes | Yes | Yes | No | Good | Yes | Fair | Unclear | Unclear |
| Campolina (2014)[13] | Yes | Yes | Unclear | Yes | Unclear | No | Unclear | Yes | Fair | Unclear | Unclear |
| Chen (2014)[20] | Yes | Yes? | Yes | Yes? | Yes | Yes | Unclear | Yes | Good | Unclear | Good |
| Hu (2019)[16] | Yes | Unclear | Yes | [NA, GBD] | NA | Unclear | NA | Unclear | Unclear | NA | NA |
| Huo (2016)[28] | Yes | Yes | Yes | No | Yes | Yes | Good | Unclear | Good | Unclear | N/A |
| Manton (1991)[4] | Yes | Yes? | Yes? | Partly | Unclear | No | Unclear | Unclear | Good | Poor | Unclear |
| Mathers (1999)[14] | Unclear | Unclear | Yes | No | Unclear | No | Unclear | Yes | Fair | Unclear | Unclear |
| Murtaugh (2011)[5] | Unclear | Unclear | Yes | Partly | Unclear | No | Good | Unclear | Good; Fair (for physical health & endurance, and mobility measures) | Poor | Unclear |
| Nusselder (1996)[15] | Yes | Yes | Yes | Partly | Yes | No | Good | Unclear | Good | Unclear | Good? |
| Public Health Canada report (2012)[8] | Yes | Unclear | Yes | Yes | Unclear | Yes? | Good | Yes | Good | Unclear | Unclear |
| Sikdar (2010)[29] | Yes | Yes | Yes | Yes | Yes | No | Unclear | Unclear | Good | Unclear | Unclear |
| Steensma (2016)[26] | Yes | Unclear | Yes | Yes | Unclear | Yes | Unclear | Yes | Good | Unclear | Unclear |
