# Supplementary material for: The impact of long-term conditions on disability-free life expectancy: A systematic review
Source: PLOS Glob Public Health. 2022 Aug 5;2(8):e0000745. doi: 10.1371/journal.pgph.0000745 (PMC10021208; doi:10.1371/journal.pgph.0000745)
Supplement: S2 Table — (DOCX) [file pgph.0000745.s007.docx]

**S2 Table.** Quality assessment for 17 longitudinal studies (adapted version of JBI checklist supplemented with Freedman criteria)

| Study (year) | Exposure measured in a valid and reliable way? (Yes if register or hospital; No if self-report only of LTC) | Confounding factors identified? | Strategies to deal with confounding factors stated? | **Width of time frame, yrs (≥8 ; 6-7 ; ≤5 years)** | **Frequency of measurement or number of time points (Annual or ≥5 times; Every 2 years or 3-4 times; >Every 2 years or 2 times)** | Outcomes measured in a valid and reliable way? | **Quality of outcome measures (Detailed self-reports; Global self-reports; none)** | Follow up complete, and if not, were the reasons to loss to follow up described and explored? (**supplement with Loss to FU (Not applicable or <5; 5-10; >10**)) | **Proxy, % (<10; 10-20; >20)** | **Missing data, % (<5; 5-10; >10)** |
| --- | --- | --- | --- | --- | --- | --- | --- | --- | --- | --- |
| Andrade (2010)[9] | No | No | No | Poor | Poor | Yes | Good | Fair | Unclear | Fair |
| Bardenheier (2016a)[6] | No | Yes | No | Good | Good | Yes | Fair | Fair | Unclear | Unclear |
| Bardenheier (2016b)[10] | No | Yes | Yes | Good | Good | Yes | Fair | Fair | Good | Unclear |
| Belanger (2002)[2] | No | No | No | Poor | Poor | Unclear | Fair | Unclear | Unclear | Unclear |
| Chiu (2019)[21] | No | Yes | Yes | Good | Good | Yes | Good | Fair | Fair | Good |
| Diehr (1998)[30] | Yes | No | No | Fair | Good | Yes | Fair | Unclear | Unclear | Fair |
| Dodge (2003)[25] | Yes | Yes | Yes | Good | Fair | Yes | Fair | Fair | Unclear | Unclear |
| Fang (2009)[22] | Yes | No | No | Poor | Poor | Yes | Fair | Fair | Unclear | Good |
| Hayward (1998)[18] | No | No | No | Fair | Fair | Yes | Good | Unclear | Unclear | Unclear |
| Jagger (2003)[31] | Partly | Yes | No | Good | Good | Yes | Fair | Unclear | Good | Unclear |
| Jagger (2007)[17] | Partly | Yes | Yes | Good | Fair | Yes | Good | Unclear | Unclear | Good |
| Liang (2020)[19] | No | No | No | Good | Fair | Yes | Fair | Unclear | Unclear | Unclear |
| Laditka (2016)[7] | No | Yes | Yes | Good | Good | Yes | Fair | Unclear | Unclear | Unclear |
| Peres (2008)[27] | No | Yes | Yes | Good | Fair | Yes | Good | Poor | Unclear | Good |
| Reynolds (2008a)[23] | No | No | No | Poor | Fair | Yes | Fair | Unclear | Fair | Unclear |
| Reynolds (2008b)[11] | No | Yes | Yes | Poor | Fair | Yes | Fair | Unclear | Fair | Unclear |
| Tareque (2019)[24] | No | No | No | Fair | Fair | Yes | Good | Poor | Good | Unclear |
