# Supplementary material for: The impact of long-term conditions on disability-free life expectancy: A systematic review
Source: PLOS Glob Public Health. 2022 Aug 5;2(8):e0000745. doi: 10.1371/journal.pgph.0000745 (PMC10021208; doi:10.1371/journal.pgph.0000745)
Supplement: S1 Methods — (DOCX) [file pgph.0000745.s008.docx]

**S1 Methods**

*Key concepts*

Life expectancy is a measure of the number of remaining years to be lived at a particular age, considering the current mortality level of a country. Health expectancy is a general term referring to the entire **class of indicators** expressed in terms of life expectancy in a defined state of health. As such, health expectancy adds a quality dimension to the quantity of life lived. Since there are many dimensions of health and ways to measure it (e.g., disability, disease, self-perceived health), there are also many health expectancies. Disability-free (or active) life expectancy is one of the most common health expectancies reported; it is based on limitations in daily activities and describes the average number of remaining years to be lived *without* disability at a certain age. Total life expectancy can be estimated by combining disability-*free* life expectancy (DFLE) and life expectancy *with* disability (DLE). Other common health expectancies are: healthy life expectancy (HLE - based on self-assessed health status), life expectancy free of specific diseases, for example dementia-free life expectancy, and health-adjusted life expectancy (HALE) where years of life are weighted by health status. Many diseases affect mortality and disability, and elimination of certain conditions may result in longer LE in poor or good health, and longer periods with or without disability. An increase in DFLE years that exceeds the increase in total LE indicates reduced number of years with disability, in which case there is an *absolute compression of morbidity*. If LE with and without disability increases and the proportion of DFLE also increases, there is a *relative compression of morbidity*. If LE with and without disability increases but the proportion of DFLE decreases, then there is a *relative expansion of morbidity* [1].
