# Supplementary material for: The impact of long-term conditions on disability-free life expectancy: A systematic review
Source: PLOS Glob Public Health. 2022 Aug 5;2(8):e0000745. doi: 10.1371/journal.pgph.0000745 (PMC10021208; doi:10.1371/journal.pgph.0000745)
Supplement: S2 Methods — (DOCX) [file pgph.0000745.s009.docx]

**S2 Methods**

*Search strategy example*

Database(s): **Ovid MEDLINE(R)** 1946 to July Week 2 2020  
Search Strategy:

| **#** | **Searches** |
| --- | --- |
| 1 | chronic disease/ or multiple chronic conditions/ |
| 2 | ((chronic* or long term or long-term) adj2 (illness* or disease* or condition*)).ti,ab,kw. |
| 3 | comorbidity/ or multimorbidity/ |
| 4 | (co-morbid* or comorbid* or multi-morbid* or multimorbid*).ti,ab,kw. |
| 5 | ((co-exist* or coexist* or co-occur* or cooccur* or concurrent* or multiple or associated or additional*) adj2 (disease* or condition* or illness* or disabilit*)).ti,ab,kw. |
| 6 | or/1-5 |
| 7 | arthritis/ or exp arthritis, rheumatoid/ or exp osteoarthritis/ or periarthritis/ or exp spondylarthritis/ |
| 8 | (arthriti* or osteoarthriti* or periarthriti* or spondylarthriti* or spondyliti* or reiter or reiter* disease).ti,ab,kw. |
| 9 | 7 or 8 |
| 10 | exp respiratory tract diseases/ |
| 11 | (respiratory adj2 (illness* or disease* or condition*)).ti,ab,kw. |
| 12 | (asthma* or bronchitis or COPD or chronic obstructive).ti,ab,kw. |
| 13 | or/10-12 |
| 14 | myocardial ischemia/ or exp angina pectoris/ or exp myocardial infarction/ or exp coronary disease/ |
| 15 | (heart disease* or heart attack* or angina*).ti,ab,kw. |
| 16 | 14 or 15 |
| 17 | exp Cardiovascular Diseases/ |
| 18 | (cardiovascular or peripheral vascular or peripheral arterial or intermittent claudication or stroke*).ti,ab,kw. |
| 19 | 17 or 18 |
| 20 | exp Cognition Disorders/ |
| 21 | (cogniti* adj2 (disorder* or dysfunction* or impair* or loss or lost)).ti,ab,kw. |
| 22 | 20 or 21 |
| 23 | diabetes mellitus/ or exp diabetes mellitus, type 1/ or exp diabetes mellitus, type 2/ |
| 24 | (diabetes or diabetic).ti,ab,kw. |
| 25 | 23 or 24 |
| 26 | exp Vision Disorders/ |
| 27 | ((visual* or vision or eyesight or sight) adj2 (disorder* or dysfunction* or impair* or loss or lost)).ti,ab,kw. |
| 28 | (blindness or partial* sight* or squint or diplopi* or amblyopi*).ti,ab,kw. |
| 29 | or/26-28 |
| 30 | dementia/ or alzheimer disease/ or exp dementia, vascular/ |
| 31 | (dementia* or Alzheimer*).ti,ab,kw. |
| 32 | 30 or 31 |
| 33 | exp Hearing Disorders/ |
| 34 | ((hearing or ear or ears) adj2 (disorder* or dysfunction* or impair* or loss or lost)).ti,ab,kw. |
| 35 | (deaf* or tinnitus).ti,ab,kw. |
| 36 | or/33-35 |
| 37 | exp Anxiety Disorders/ or exp Mood Disorders/ |
| 38 | exp "Bipolar and Related Disorders"/ |
| 39 | exp "Schizophrenia Spectrum and Other Psychotic Disorders"/ |
| 40 | (mental health or mental illness* or anxiety or anxious* or depression or bipolar or schizophreni* or psychosis or psychotic).ti,ab,kw. |
| 41 | or/37-40 |
| 42 | 6 or 9 or 13 or 16 or 19 or 22 or 25 or 29 or 32 or 36 or 41 |
| 43 | ((health* or active* or disability*) adj2 life expectanc*).ti,ab,kw. |
| 44 | ((disabilit* or disable*) adj3 (expectanc* or year*)).ti,ab,kw. |
| 45 | 43 or 44 |
| 46 | 42 and 45 |
| 47 | limit 46 to (english language and humans and yr="2007 -Current") |
| 48 | (comment or editorial or letter or news or newspaper article).pt. |
| 49 | 47 not 48 |
