# Supplementary material for: The impact of long-term conditions on disability-free life expectancy: A systematic review
Source: PLOS Glob Public Health. 2022 Aug 5;2(8):e0000745. doi: 10.1371/journal.pgph.0000745 (PMC10021208; doi:10.1371/journal.pgph.0000745)
Supplement: S3 Methods — (DOCX) [file pgph.0000745.s010.docx]

**S3 Methods**

*Quality assessment*

All but four studies [2-5] reported valid and reliable criteria to measure the outcomes that were almost exclusively self-reported. Only a small number of studies (28%) measured disability with a single item (e.g., asking whether the participant has any limitation in a number of ADLs), and the rest used a combination of activity limitation indicators. Several studies identified potential confounding factors, yet only a few were able to control for such factors in their analysis (e.g., presence of multimorbidity). The proportion of proxy interviews and missing data was underreported, and marked as unclear, in 72% of the studies.

Among the longitudinal studies, interview methods were identical across the repeated measurements in all but one study [6] where one of the three activity limitation indicators used differed in an early wave of the cohort. About half of the studies received a fair rating for the frequency of measurement or number of time points of the analysis (translating as every 2 years or 3-4 times), and a good rating for a time frame exceeding 8 years (see S1 Table, S2 Table*).*
