# Supplementary material for: The impact of long-term conditions on disability-free life expectancy: A systematic review
Source: PLOS Glob Public Health. 2022 Aug 5;2(8):e0000745. doi: 10.1371/journal.pgph.0000745 (PMC10021208; doi:10.1371/journal.pgph.0000745)
Supplement: S2 Results — (DOCX) [file pgph.0000745.s012.docx]

**S1 Results**

*Effect of LTCs on health expectancy (narrative syntheses)*

*Diabetes*

Nine studies assessed the impact of diabetes at age 45 [2], 55 [7, 8], 60 [6, 9, 10], and 70 years [11] (Table 2). At age 45, diabetes had a strong impact on both mortality and disability. The difference between those without and those with diabetes was (in absolute terms) greater among women than men (LE difference, men: 5.6 years; women: 10.7 years). Diabetes was associated with an even greater impact on DFLE (DFLE difference, men: 13.0 years; women: 14.1 years) suggesting that if eliminated it could be expected to result in compression in disability [2]. Estimates reported for individuals at age 55 by two studies suggested a similar pattern, although differences between those without and with diabetes were generally smaller [7, 8]. At age 60, the loss of LE for those with diabetes compared to those without was on average higher for men than women, and the majority of the years lost were disability-free years [6, 9, 10], again highlighting the disabling effect of diabetes. Supporting this further, Campolina and colleagues [12, 13] showed that the hypothetical elimination of diabetes at age 60 would result in significant gains in DFLE for both men and women and this gain would exceed the accompanying increase in LE (Table 2), particularly among women who would also experience a strong reduction in DLE years. By age 70, both men and women with diabetes are expected to have shorter LE and DFLE than those without the condition, and to spend greater percentage of remaining life with disability compared to those without diabetes [11]. The majority of the studies therefore concluded that elimination of diabetes would result in a compression of disability.

*Cardiovascular disease*

Twelve studies (seven cross-sectional and five longitudinal) assessed the impact of cardiovascular diseases including ischaemic heart disease, heart attack, and peripheral vascular disease on health expectancy. Estimates from a US study assessing the impact of multiple conditions at age 55 and stratified by race/ethnicity [7], showed great reductions in LE and even greater loss in DFLE years for those with heart disease compared to those without (Table 4). African American men with heart disease showed the highest percentage of remaining life with disability (40%) from age 55 compared to white men and women with or without the LTC [7]. Estimates from the 2000 SABE study indicated that elimination of heart disease would lead to substantial increase in LE at age 60 particularly among men; in women these years corresponded to a greater gain in DFLE of 8.6 years and a reduction of 5.8 years in DLE but in men elimination would translate to a big increase in DFLE (7.7 years) with a small increase in DLE (0.12 years). Ten years later, analysis of the 2010 wave of SABE [13] showed the same pattern for men and women with increased LE and DFLE and reduced DLE for both sexes after disease elimination. The proportion of disability-free years resulting from disease elimination also increased between waves [12, 13]. Studies reporting estimates for heart disease elimination at age 65 [3, 14, 15], indicated it would result in moderate increases in LE (range in LE gain: 1.1 to 3.1 years) which would however be greater than the expected increases in DFLE or HALE, suggesting a relative expansion of disability. In one study reporting trends in Chinese elderly from 1990 to 2016, potential gains in DFLE after elimination of cardiovascular diseases nearly doubled with a slightly higher increase for women (no LE estimates reported) [16]. Very small reductions in LE and almost no difference in the duration of ADL- or IADL-disability for those without and with the condition was reported for the effect of heart attack [5]. In a longitudinal analysis from the MRC CFAS study [17], the largest proportion of reductions in LE was felt in terms of disability-free years in individuals with coronary heart disease or peripheral vascular disease, when compared to those without each of the LTCs (Table 4). However, results from this study suggest that elimination of heart disease would likely result in compression of disability in women and elimination of peripheral vascular disease would likely result in compression of disability in men [17].

Estimates from the AHEAD study [11] in the US showed that at age 70, the LE difference between those without and those with heart disease was 3.2 years for men and 4.2 years for women, and the majority of years lost were disability-free years (Table 4). Women with heart disease were expected to live longer with disability than men with heart disease (% DLE, women: 37.3%, men: 22.5%) [11]. Elimination of heart disease in another US study [18] would result in a 3.1-year gain in LE for men and a 3.9-year for women. However, elimination would not affect men and women equally: most of the gain in LE would be felt as an increase in DFLE for men but for women the majority of that gain would be added years with disability [18].

*Hypertension*

Four cross-sectional [8, 12-14] and two longitudinal [7, 19] studies assessed the effect of hypertension on health expectancy at various ages (55, 60 and 65) (Table 5). Analyses from two studies carried out in Brazil [12, 13] suggested that elimination of hypertension at age 60 would lead to greater gain in DFLE in relation to the gain in LE indicating a compression of disability for both sexes, but estimates were higher for women compared to men (e.g. proportion of life lived disability-free after elimination, men 17.9% and women 38.4%) [13]. Two studies reporting HALE as the outcome at ages 55 and 65 also supported greater gain in HALE in relation to the gain in LE after elimination of hypertension although the estimated gains were smaller ranging from 2.7 years at age 55[8] to 0.13 years at 65.[14] In a US study stratifying results by sex and race/ethnicity [7], hypertension was among the individual LTCs examined responsible for the greatest reduction in LE at 55, especially in white women. Compared with white women who did not have hypertension, those with the condition lived 13.5 fewer years with a life expectancy of 73.8 (70.8–76.8), and a larger proportion of remaining life with disability (38.9% vs. 12.2%) [7]. By contrast, LE and DFLE at age 65 was largely similar in persons without and with hypertension (less than 1 year difference) in the Taiwan Longitudinal Study on Aging [19].

*Cerebrovascular disease*

Six studies [3, 12-14, 18, 20] evaluated the impact of cerebrovascular diseases on health expectancy focusing primarily on gain in LE and DFLE/HALE after the hypothetical elimination of the condition, while one of the studies reported only the contribution of the LTC to disability burden [20] (Table 6). Cerebrovascular diseases were among the five individual diseases with the highest prevalence and most disabling impact for disability burden in China [20] with life expectancy with disability at age 60-69 estimated at 0.45 and 0.39 years for men and women respectively. Based on analysis of the 2000 SABE data in Brazil [12], elimination of cerebrovascular disease would lead to substantial gain in LE at age 60 particularly among men; in women these years corresponded to a gain of 6.83 years in DFLE and a reduction of 4.39 years in DLE but in men elimination would translate to increases in both DFLE (6.47 years) and DLE (0.86 years). Ten years later, analysis of the 2010 wave of SABE [13] showed the same pattern for men and women with increased LE and DFLE and reduced DLE for both sexes after disease elimination. Estimates from two studies at age 65 [3, 14] and one study at 70 [18] also indicated gains in LE after disease elimination (although less than 1 year), with some increase in DLE but the highest proportion added to DFLE [3, 18] or HALE [14].

*Stroke*

One cross-sectional [5] and five longitudinal [7, 11, 17, 21, 22] studies assessed the effect of stroke on health expectancy (Table 7). Murtaugh and colleagues [5] showed that both men and women with stroke had greater than average LE at age 65, and the condition was associated with 0.2 to 0.5 years longer duration of disability than those without stroke. One study based on data from two Chinese cohorts reported temporal comparisons for 1992 and 2000 [22]. The comparison showed that people with stroke in the later cohort (2000) were expected to live longer, spend more years disability-free and fewer years with disability compared to those with stroke in 1992. However, stroke remained an important cause of disability, and disability status at baseline played an important role. At age 65, people with stroke and baseline disability had shorter LE by 20-40% with disability-free years reduced by up to 90%, whereas LE and DFLE patterns were similar for those without baseline disability regardless of stroke status [22]. A similar pattern with greater reduction in DFLE than LE was estimated in the remaining four longitudinal studies. Estimates from the US study assessing the impact of multiple conditions at age 55 and stratified by race/ethnicity [7], indicated devastating effects of stroke on LE and DFLE particularly for women with a loss of more than 10 and 20 years respectively. Studies at older ages (65 and 70) from the UK [17], US [11] and Japan [21] also showed that those who have experienced stroke could expect on average a reduction in overall LE and DFLE by almost 50%, compared to those who had not had a stroke, especially when initially classified as ADL/IADL disabled [21].

*Cancer*

Ten studies (seven cross-sectional [3, 8, 12-16] and three longitudinal [2, 11, 18]) evaluated the impact of cancer (Table 8). As expected, cancer had a major impact on LE and a lesser impact on DFLE reduction. Cancer impact on mortality at age 45 was so great (>10 years loss) that individuals with cancer were expected to live more years disability-free and fewer years with disability than those without the condition [2]. Similar patterns were reported at older ages again with greater reduction in LE than HALE [8] or DFLE [11] for both sexes. However, by age 70 the difference in DFLE and DLE years between those without and with cancer had narrowed, and men with cancer had significantly reduced proportion of remaining life disability-free compared to their counterparts without cancer (e.g., % DFLE without cancer vs with cancer: 80% vs. 76%). In women, the proportion of remaining life disability-free was lower than in men but similar in those with and without cancer (~70%) [11]. Seven of the studies assessed the impact of cancer elimination with mixed findings. An evaluation of survey data from 2000 in Brazil [12] showed that elimination of cancer at 60 would increase LE and DFLE and decrease years lived with disability in women suggesting a compression of disability but would result in increased DLE in men (signifying expansion of disability). However, the pattern for men changed in the later studied cohort (in 2010), indicating a reduction in DLE after cancer elimination in both sexes [13]. At age 65, elimination of cancer would lead to more modest increases in LE (between 1.07 and 2.7 years) accompanied by a small gain in DFLE [3, 15] and HALE [14] but this did not always translate into increased proportion of remaining life disability-free for men [14, 15]. In one study reporting estimates at age 70, the elimination of cancer would result in 1.6 years gain in LE for men and 1.2 years for women, with most of the gain added to DFLE [18]. In the only study reporting trends in Chinese elderly from 1990 to 2016, DFLE generally increased along with potential gains in DFLE after elimination of cancer with a slightly higher increase in men than women (no LE estimates reported) [16].

*Arthritis*

Five studies [2, 5, 7, 17, 23] examined the effect of arthritis and two additional studies [14, 20] reported specifically on the effect of osteoarthritis and rheumatoid arthritis on health expectancy (Table 9). In all studies reporting relevant estimates, arthritis was associated with a small loss of LE and it was generally greater for women. One of the studies stratifying analysis by race/ethnicity estimated greater LE loss in absolute terms for white men and women compared to African Americans at age 55 (men: 7.3 *vs.* 0.4 years, women: 9.6 *vs*. 4.8 years) [7]. However, arthritis had a greater effect on DFLE than LE consistently across studies and the reduction was again greater for women, ranging from a decade or more in cohorts at age 45 and 55 to around 2 years at age 70. In the study by Murtaugh and colleagues [5] assessing the impact of multiple LTCs at age 65 including diabetes, heart disease and COPD which are also included in this review, arthritis was associated with the highest duration of disability in women for all four types of disability examined and DFLE shorter than the overall average. Osteoarthritis was identified as one of the most important diseases for disability burden at age 60 in China among several LTCs assessed, although DLE estimates are small (DLE, men: 0.27; women: 0.48) compared to other studies in this LTC group. Based on calculations using data from the 1993 Australian survey of disability [14], elimination of osteoarthritis in women would lead to one of the top three gains in HALE at age 65 (HALE gain: 0.85 years) following IHD, with a smaller gain for elimination of rheumatoid arthritis (HALE gain: 0.11 years). Elimination of osteoarthritis was also among the top ten greatest gains in HALE at age 65 for men in the same study (HALE gain: 0.28 years) [14].

*Sensory loss*

Four studies [14, 17, 20, 24] reported the impact of hearing loss or impairment of which two [17, 24] also included the impact of visual impairment on LE and DFLE at age 60 or 65 (Table 10). One study [20] reported only life expectancy with disability for those with hearing loss at age 60 of around 1.5 years without additional information on LE. As expected, hearing loss/impairment had a small impact on LE (range: 0.0-0.5 years), and a greater impact on DFLE reduction (or HALE) ranging from 0.5 to 2.1 years in men and 0.16 to 1.2 years in women [14, 17]. A similar pattern was observed for visual impairment although the impact on LE and DFLE was slightly greater than that of hearing impairment and for women (LE reduction, range: 0.9-2.6 years; DFLE reduction, range: 2.0-4.4 years) [14, 17]. Those with both impairments at age 60 as examined in the study by Tareque and colleagues [24], could expect shorter LE than those with neither condition (4.2 less years), a loss of 6.6 years in DFLE, and a 15% increase in the proportion of years with ADL limitations (i.e., 2.4 years).

*Dementia & cognitive impairment*

Five studies examined the impact of dementia [4, 5, 25] or cognitive impairment [7, 17], although these were at different ages and using different disability measures and outcomes (Table 11). Even though increasing age is associated with greater risk of cognitive impairment and dementia, people with these conditions generally had shorter LE than those without, and the loss in LE was, in absolute terms, greater for women than men. Dementia and related conditions appeared to have a greater effect on DFLE than LE. Analysis of an older US cohort based on 27 limitation measures and several types of disability showed a small gain in DFLE after the hypothetical elimination of dementia at 65 (men: 0.22 years, women: 0.48 years) [4] although LE estimates were not reported. In another study, the projected period of difficulty with four types of functioning including ADLs and IADLs at age 65 was nearly twice as long for those with dementia compared to those without, exceeding 4 and 5 years for IADL disability for men and women with dementia respectively [5]. Memory impairment at age 55 significantly reduced DFLE in white and African American men and women in a US study stratified by race/ethnicity [7] compared to those without any conditions, ranging from 7 to 8.6 years in men and 9 to 14 years in women. Cognitive impairment also reduced DFLE at age 65 by about 4 years in men and women in a UK cohort [17]. Individuals with Alzheimer’s disease at age 70 had a reduction in DFLE (none or minimal disability) of approximately 40% compared to those without the condition (DFLE, men: 5.0 *vs.* 12.1 years; women: 4.9 *vs*. 13.4 years) in a longitudinal US study [25].

*Depression*

One cross-sectional [26] and three longitudinal studies [7, 11, 27] examined the impact of depression on LE and DFLE (or HALE) at ages 55, 65 or 70 (Table 12). Depression and emotional problems had a greater impact on reduction of DFLE (or HALE in one study [26]) than LE for both men and women, although differences in DFLE between men and women were not consistent across studies. Absolute reduction in total LE and DFLE were greater for white women with depression compared to African American women in a US study stratified by race/ethnicity; the opposite was observed for men, with greater loss both in LE and DFLE for African American men with depression compared to white men. Noting that studies reported estimates at different ages, the impact of depression on LE reduction varied from 0.8 to 7.3 years and on DFLE reduction from 1.4 to 12.4 years.
