# Supplementary material for: The impact of long-term conditions on disability-free life expectancy: A systematic review
Source: PLOS Glob Public Health. 2022 Aug 5;2(8):e0000745. doi: 10.1371/journal.pgph.0000745 (PMC10021208; doi:10.1371/journal.pgph.0000745)
Supplement: S1 References — (DOCX) [file pgph.0000745.s013.docx]

**S1 References**

1. Jagger C, Crimmins EM, Saito Y, Yokota RTDC, Van Oyen H, Robine J-M, editors. International handbook of health expectancies: Springer Nature; 2020.

2. Belanger A, Martel L, Berthelot J-M, Wilkins R. Gender differences in disability-free life expectancy for selected risk factors and chronic conditions in Canada. Journal of women & aging. 2002;14(1-2):61-83.

3. Brønnum-Hansen H, Juel K, Davidsen M. The burden of selected diseases among older people in Denmark. Journal of aging and health. 2006;18(4):491-506.

4. Manton KG, Stallard E. Cross-sectional estimates of active life expectancy for the U.S. elderly and oldest-old populations. Journal of gerontology. 1991;46(3):S170-82.

5. Murtaugh CM, Spillman BC, Wang XD. Lifetime risk and duration of chronic disease and disability. Journal of Aging & Health. 2011;23(3):554-77.

6. Bardenheier BH, Lin J, Zhuo X, Ali MK, Thompson TJ, Cheng YJ, et al. Compression of disability between two birth cohorts of US adults with diabetes, 1992-2012: a prospective longitudinal analysis. The Lancet Diabetes & Endocrinology. 4(8):686-94. PubMed PMID: rayyan-95875019.

7. Laditka JN, Laditka SB. Associations of multiple chronic health conditions with active life expectancy in the United States. Disability & Rehabilitation. 2016;38(4):354-61.

8. Public Health Agency of Canada Steering Committee on Health-Adjusted Life E. Report summary--Health-Adjusted Life Expectancy in Canada: 2012 Report by the Public Health Agency of Canada. Chronic Diseases and Injuries in Canada. 2012;33(2):103.

9. Andrade FC. Measuring the impact of diabetes on life expectancy and disability-free life expectancy among older adults in Mexico. Journals of Gerontology Series B-Psychological Sciences & Social Sciences. 65(3):381-9.

10. Bardenheier BH, Lin J, Zhuo X, Ali MK, Thompson TJ, Cheng YJ, et al. Disability-Free Life-Years Lost Among Adults Aged >=50 Years With and Without Diabetes. Diabetes Care. 39(7):1222-9.

11. Reynolds SL, Haley WE, Kozlenko N. The impact of depressive symptoms and chronic diseases on active life expectancy in older Americans. American Journal of Geriatric Psychiatry. 2008;16(5):425-32.

12. Campolina AG, Adami F, Santos JL, Lebrao ML. Effect of eliminating chronic diseases among elderly individuals. Revista de Saude Publica. 2013;47(3):514-22.

13. Campolina AG, Adami F, Santos JL, Lebrao ML. Effect of the elimination of chronic diseases on disability-free life expectancy among elderly individuals in Sao Paulo, Brazil, 2010. Ciencia & Saude Coletiva. 2014;19(8):3327-34.

14. Mathers CD. Gains in health expectancy from the elimination of diseases among older people. Disability and rehabilitation. 1999;21(5-6):211-21.

15. Nusselder WJ, van der Velden K, van Sonsbeek JL, Lenior ME, van den Bos GA. The elimination of selected chronic diseases in a population: the compression and expansion of morbidity. American journal of public health. 1996;86(2):187-94.

16. Hu X, Sun X, Li Y, Gu Y, Huang M, Wei J, et al. Potential gains in health-adjusted life expectancy from reducing four main non-communicable diseases among Chinese elderly. BMC Geriatrics. 19(1):16.

17. Jagger C, Matthews R, Matthews F, Robinson T, Robine JM, Brayne C, et al. The burden of diseases on disability-free life expectancy in later life. Journals of Gerontology Series A-Biological Sciences & Medical Sciences. 2007;62(4):408-14.

18. Hayward MD, Crimmins EM, Saito Y. Cause of death and active life expectancy in the older population of the United States. Journal of Aging and Health. 1998;10(2):192-213.

19. Liang C-C, Hsu W-C, Tsai Y-T, Weng S-J, Yang H-P, Liu S-C. Healthy Life Expectancies by the Effects of Hypertension and Diabetes for the Middle Aged and Over in Taiwan. International Journal of Environmental Research & Public Health [Electronic Resource]. 2020;17(12):18.

20. Chen H, Wang H, Crimmins EM, Chen G, Huang C, Zheng X. The contributions of diseases to disability burden among the elderly population in China. Journal of Aging & Health. 2014;26(2):261-82.

21. Chiu CT, Yong V, Chen HW, Saito Y. Disabled life expectancy with and without stroke: a 10-year Japanese prospective cohort study. Quality of Life Research. 2019;28(11):3055-64.

22. Fang XH, Zimmer Z, Kaneda T, Tang Z, Xiang MJ. Stroke and active life expectancy among older adults in Beijing, China. Disability & Rehabilitation. 2009;31(9):701-11.

23. Reynolds SL, McIlvane JM. The impact of obesity and arthritis on active life expectancy in older Americans. Obesity. 2008;17(2):363-9.

24. Tareque MI, Chan A, Saito Y, Ma S, Malhotra R. The Impact of Self-Reported Vision and Hearing Impairment on Health Expectancy. Journal of the American Geriatrics Society. 2019;67(12):2528-36.

25. Dodge HH, Shen C, Pandav R, DeKosky ST, Ganguli M. Functional transitions and active life expectancy associated with Alzheimer disease. Archives of neurology. 2003;60(2):253-9.

26. Steensma C, Loukine L, Orpana H, McRae L, Vachon J, Mo F, et al. Describing the population health burden of depression: health-adjusted life expectancy by depression status in Canada. Health Promotion and Chronic Disease Prevention in Canada. 2016;36(10):205-13.

27. Pérès K, Jagger C, Matthews FE. Impact of late‐life self‐reported emotional problems on Disability‐Free Life Expectancy: results from the MRC Cognitive Function and Ageing Study. International Journal of Geriatric Psychiatry: A journal of the psychiatry of late life and allied sciences. 2008;23(6):643-9.

28. Huo L, Shaw JE, Wong E, Harding JL, Peeters A, Magliano DJ. Burden of diabetes in Australia: Life expectancy and disability-free life expectancy in older adults with diabetes. Diabetes. 2016;65:A409.

29. Sikdar KC, Wang PP, MacDonald D, Gadag VG. Diabetes and its impact on health-related quality of life: a life table analysis. Quality of Life Research. 2010;19(6):781-7.

30. Diehr P, Patrick DL, Bild DE, Burke GL, Williamson JD. Predicting future years of healthy life for older adults. Journal of Clinical Epidemiology. 1998;51(4):343-53.

31. Jagger C, Goyder E, Clarke M, Brouard N, Arthur A. Active life expectancy in people with and without diabetes. Journal of public health medicine. 2003;25(1):42-6.
