# Supplementary material for: The impact of long-term conditions on disability-free life expectancy: A systematic review
Source: PLOS Glob Public Health. 2022 Aug 5;2(8):e0000745. doi: 10.1371/journal.pgph.0000745 (PMC10021208; doi:10.1371/journal.pgph.0000745)
Supplement: Attachment — Submitted filename: RESPONSE TO REVIEWERS_IL_CJ_IL.docx [file pgph.0000745.s014.docx]

# RESPONSE TO REVIEWERS

*PLOS Global Public Health Decision: Revision required [PGPH-D-21-01155]: The impact of long-term conditions on disability-free life expectancy: a systematic review*

We thank the reviewers for their comments on the manuscript and we have addressed each of these below. In addition to the review comments, we have also addressed the formatting issues raised by the editorial team. These are amended in the manuscript, and we detail that amendment at the end of this document.

We hope that the responses are satisfactory, and we would welcome a positive review for publication.

**Reviewer #1:**

**Line number- 29, 132: Please, mention the name of month along with the year 2007.**

*Now added in manuscript, lines 29 and 152.*

**Line number- 596, 625: Homogeneity should be maintained during writing references. As because all the references didn't contain doi number, you can omit the doi numbers.**

*References were entered using a reference management software with doi numbers automatically assigned to each reference where available. We have however deleted doi numbers assigned by the software used for screening since they are not needed.*

**Reviewer #2**

**Is the term ‘long-term conditions’ (LTCs) used is standard or is it used for this current study purpose; if so definition to be given**

*The term ‘Long Term Condition (LTC)’ is a standard term used in the UK and across the world. Its defined as: ‘a condition that cannot, at present, be cured but is controlled by medication and/or other treatment/therapies.’*

*We recognise that some readers of this paper may be familiar with the term and so we have incorporated a definition into the manuscript. (line 103)*

**What is the PICO question for this review?**

*The PICO framework is often used to structure the reporting of inclusion and exclusion criteria for reviews of interventions. Our review does not examine the effect of*

*interventions, however, a statement of the review objectives is provided in lines 135-138, and the eligibility criteria are outlined in the section under the same heading.*

**It was very wage LTCs; it need to specifically mention that what diseases included in the review; time period (last ten or twenty years), countries (developed and developing); whether unpublished literature is included; other than English included or not**

*The included conditions and the rationale for their inclusion in the review are reported under the section ‘Eligibility criteria’ lines 161-178. There were no restrictions other than English language (line 152); data sources and details of the searches can be found in lines 148-159.*

**What is the outcome parameter is not clear in the data extraction section**

*Reported outcomes tended to differ across studies. Details of relevant outcomes that were considered are reported in lines 172-178 (text below)*

*“Studies were included if they measured the effect of any of the above LTCs on health expectancy outcomes, primarily disability-free, active or healthy life expectancy reported at age 50 and older compared to individuals without the LTC. Studies reporting estimates at birth only were excluded. Outcomes reported as gains in DFLE or HALE with and without a specific LTC (or multimorbidity) and estimates from projections of DFLE were also eligible. Total life expectancy was eligible as an outcome only when reported alongside the main outcomes (DFLE, ALE, HALE).”*

**Data extraction: Provide details of what data were extracted. What are the statistical tools used? any software used for search and analysis?**

*All relevant information for data extraction is reported in lines 190-207, and for data synthesis and analysis in lines 214-229.*

**If full paper not available what action was taken to get full paper for review; any missing information; how the missing information is managed; whether contacted author to provide information?**

*Authors of four papers were contacted for clarification or additional relevant information; please see line 198-199. Due to available capacity and timescale of the project, unsuccessful internet searches to obtain the full text of potentially relevant papers meant they were excluded, as listed in figure 1.*

**How the quality of the evidence assessed?**

*Please see lines 209-213 and S3 Methods text for reporting of this stage.*

**How risk of bias assessed; any standard check list used**

*The quality assessment we followed incorporates risk of bias assessment. Please see line 225 and S3 Methods text for reporting of this stage.*

**The statement “The funnel plot indicated potential publication bias. Results of the Egger test for small-study effects suggested this is unlikely to be problematic. Meta-analysis of LE estimates for the above four studies was not possible due to insufficient data” what authors want to convey from the results. If it is biased is it possible to remove or reduce bias and recalculate.**

*It is likely that the tests accompanying the meta-analysis were underpowered to detect bias, and the high heterogeneity observed could not be further explored due to the small number of included studies. We discuss this in the Discussion section, lines 459-462.*

**Overall authors need to simplify or make it non-statistician to understand the results.**

*We agree that this is a complex paper but, as this is a systematics review, we think it is important to provide results for all the LTCs rather than write separate papers. We believe that the use of subheadings helps guide the reader for each LTC. However, we have now sub-headed Data extraction and Quality Assessment separately to make that clearer.*

*The only statistical results provided are for the meta-analysis and these have followed standard reporting methods.*

**Hu et al.2019 reported estimates for two population samples; is it from different region/ area; it seems to be same study for respiratory diseases also. It is calculated for age at 65 years; what about other age group**.

*Indeed Hu et al. reported estimates for two population samples; one for year 1990 and the other for year 2016 based on data from the Global Burden of Disease Study 2016 for China (please see Table 1). The study examined multiple LTCs which are relevant to the systematic review (please see Tables 2,3,4,8). We included studies reporting health expectancy outcomes at age 50 or older and, where multiple ages are provided, we report results at age 65 if available (please see Data Extraction section).*

**Ranking graphs needs explanation; it too much of information in one graph**

*Graphs are used to show the overall picture of the ranking of the studied LTCs, with clarifications provided in the form of a narrative summary, please see lines 384-405.*

**Reviewer #3:**

**Does the term “disability free life-expectancy” that you used is synonymous to the term “Healthy life expectancy”. If yes, then please use the term healthy life expectancy as this is common and easily understandable. If not, please write their differences in the introduction section.**

*Disability-free Life Expectancy is not synonymous to Healthy Life Expectancy. We have added reference to S1 Methods where the full definitions of disability-free and healthy life expectancy as well as the overarching term Health Expectancy (Introduction lines 113).*

**I am thinking if you can split this paper to two or more independents papers. You included many variables. Though I appreciate your efforts and hard work, however, because of such higher number of variables this paper did not end with a strong message for the areas of improvement.**

*We had initially discussed whether this was one or more than one paper. However, though the number of LTCs included is large, it is unclear how they could be split into meaningful groups and it would make much of the papers duplicated. We felt it was important to be able to see the effect of each of the LTCs together than to artificially chop them up into separate papers.*

**Abstract 1. The aims should be revised.**

*It is unclear from this comment exactly what the reviewer is unhappy with.*

**Abstract 2. What is health expectancy? Please see my overall comment 1 and changes it accordingly.**

*See response to first issue for this reviewer.*

**Abstract 3. You search literature for 2007 to 2020. Why 2007? Any specific reason for this? If yes, please mention. Moreover, your search and analysed should be updated to cover papers published from July 2020 (or April 8, 2021) to now- it is almost 1.5 years and I think some papers in this review areas have been published that should be included in this review.**

*We have now added in (lines 153-154) the reason for the choice of 2007. However we also included earlier papers from searching reference lists of publications. The authors include academics who have been integral to the International Network on Health Expectancy and the Disability Process since the early 1990s and there had been very little on the effect of LTCs on health expectancy prior to 2007.*

*It is unreasonable for the reviewer to suggest there should be no time gap in which to process the publications found for quality assessment and include them in the paper. We increased the time covered until April 2021 but required the time until Nov 2021 when the paper was submitted to process the information and write the paper.*

**Abstract 4. Methods and findings section is now much informative. This is unusual and reader may not love this. This should be revised.**

*We have included the information we think necessary and have included further details in supplementary material.*

**Abstract 5. It is methodologically wrong if you pooled (through meta-analysis) effects of different outcomes together, for instance, if you pool the effects of depression and diabetes on DFLE.**

*We refer the reviewer to the methods specifically lines 214-229. We did NOT pool the effect of different outcomes or LTCs.*

**Abstract 6. I am not sure whether you used fixed effect of random effect meta-analysis. This should be clarified in the methods section of the abstract.**

*We refer the reviewer to line 222 in the Methods where we clearly state we used a random effects meta-analysis. We have now added ‘random effects’ to the Abstract (line 44)*

**Abstract 7. Conclusion section should be revised. Currently, it does not fully support your results. I am also do not believe you study is first of its kind. Please revised this sentence.**

*We are unclear what the reviewer wishes us to change in the conclusion. We are also unsure, given the lack of a reference, which other studies have assessed the impact of LTCs on Health Expectancy – however, in case the reviewer has in mind the GBD studies, we have amended this sentence to read ‘both HE and LE’ as the GBD studies cannot infer compression or expansion.*

**Author’s summary - Line 66 should be revised.**

*We do not see anything incorrect in line 66 and are unclear what the reviewer wishes us to revise.*

**Author’s summary - The section “What do these findings mean” should be revised. This is now not eye catching.**

*We have revised this section (see lines 60-73)*

**Introduction - You introduction section should be revised to make it directable for your study. Currently, this is poorly justifying the need of this study.**

*We feel the introduction does set the scene for our study spelling out the increase in prevalence of LTC, their disabling impact, and the need to quantify systematically which conditions have the greatest effect on DFLE or LE in order to reduce disability burden.*

**Methods - You need to clarify whether you included gray literature and unpublished or not.**

*We have added a sentence to show that we only included grey literature or unpublished literature if found through reference lists.*

**Methods - The aim you write here is different from what you write in the abstract and the introduction section.**

*The aims differ only in terms of whether we have DFLE or other HEs. We have now made this clearer (Methods line 165-6) but the main aim was always to quantify the impact of LTCs on DFLE and LE but we included other HE terms to ensure that we did not miss any studies.*

**Methods - Details are needed in explaining how you choose long-term conditions.**

*In fact, the conditions were chosen mostly to correspond to an update of the 2007 paper already referred to (reference 41). This new paper has now been accepted for publication by PLOS Medicine. The only exception is cancer which was not included in either of the papers but which we felt was important to include.*

**Methods - I am not sure about the priority age you mentioned in line 170. Previously you reported you considered age 50. Please be consistent.**

*We were interested in studies of older ages so considered only studies reporting DFLE and LE at ages 50 or above. However when extracting data, if DFLE was presented at multiple ages we noted at which ages but extracted estimates at age 65 as a priority (most studies report health expectancy estimates at age 65). Please note though we did know if data were present for other ages.*

**Results - Reference list should be comprehensively searched. This is unlikely that you searched and found only one paper.**

*We did comprehensively search reference lists. That only 29 DFLE-related and seven DALY-related were eligible for inclusion, it is not surprising that searching the reference lists found only one further paper.*

**Results - I am wondering how and why you included papers spanning between 1991 and 2020 that you mentioned in line 215. You mentioned previously that you considered year 2007 to 2021.**

*See response to Abstract 3. Earlier.*

**Results - I am confused about your updated search and its result you mentioned in Figure 1. This is fully unlikely that between July 2020 to April 2021, no study other than the studies you included was published.**

*We identified 9 further studies from the updated search – see Figure 1.*

**Results - Trim and fill estimate should be performed to add potential number of missing studies.**

*We have addressed potential publication bias in the meta-analyses by calculating funnel plots. However, given there were barely enough studies to perform a meta-analysis it would be impossible to perform a trim and fill.*

**Discussion and conclusion section should be revised in line with the results. The present version is better, however, there is a space for further improvement.**

*We are unclear where the discussion is not in line with results and where further improvements may be made.*

**Changes requested by the editor to formatting**

**Please modify the formatting of your Author Summary so that it is not broken into sections.**

*We have amended this section to reflect the changes requests. This section is no longer broken into section.*

**Please provide separate figure files in .tif or .eps format only and remove any figures embedded in your manuscript file. Please ensure that all files are under our size limit of 20MB.**

*We have removed all figures from the manuscript and have uploaded them as .tif files*

**We notice that your supplementary figures and tables are included in the manuscript file. Please remove them and upload them with the file type 'Supporting Information'. Please ensure that all Supporting Information files are included correctly and that each one has a legend listed in the manuscript after the references list.**

*We have separated the supporting information form the main manuscript and have uploaded this as a separate document.*

**Please amend your detailed Financial Disclosure statement. This is published with the article, therefore should be completed in full sentences and contain the exact wording you wish to be published.**

**State the initials, alongside each funding source, of each author to receive each grant.**

*We have amended the financial disclosure state in the manuscript (line 505) and included the initials of the grant holders*
